# A New Protocol for the Synthesis of New Thioaryl-Porphyrins Derived from 5,10,15,20-Tetrakis(pentafluorophenyl)porphyrin: Photophysical Evaluation and DNA-Binding Interactive Studies

**DOI:** 10.3390/molecules23102588

**Published:** 2018-10-10

**Authors:** Patrícia Foletto, Fabiula Correa, Luciano Dornelles, Bernardo A. Iglesias, Carolina H. da Silveira, Pablo A. Nogara, João B. T. da Rocha, Maria A. F. Faustino, Oscar E. D. Rodrigues

**Affiliations:** 1 Departamento de Química, Universidade Federal de Santa Maria, Santa Maria-RS 97105-900, Brazil; patriciafoletto@hotmail.com (P.F.); fabiulacorrea11@gmail.com (F.C.); ldornel@gmail.com (L.D.); 2 Laboratório de Bioinorgânica e Materiais Porfirínicos, Departamento de Química, Universidade Federal de Santa Maria–UFSM, Santa Maria–RS 97105-900, Brazil; carolinahahndasilveira@gmail.com; 3 Laboratório de Bioquímica Toxicológica, Departamento de Bioquímica e Biologia Molecular, Univerisidade Federal de Santa Maria, Santa Maria–RS 97105-900, Brazil; pbnogara@gmail.com (P.A.N.); jbtrocha@yahoo.com.br (J.B.T.d.R.); 4 QOPNA and Department of Chemistry, University of Aveiro, Aveiro 3810-193, Portugal; faustino@ua.pt

**Keywords:** thioaryl-porphyrins, disulfides, DNA interaction, photophysical characterization

## Abstract

A new protocol for the preparation of thioaryl-porphyrins is described. The compounds were prepared from different disulfides employing NaBH_4_ as a reducing agent. The methodology allowed the preparation of four different thioaryl-porphyrins in very-good to excellent yields under soft conditions, such as short reaction times and smooth heating. Additionally, the photophysical properties of new compounds were determined and experimental and theoretical DNA interactions were assessed.

## 1. Introduction

Porphyrins are a useful class of aromatic macrocycles. These compounds have diverse applications, including medicinal [1], electronic devices [2], energy conversion [3], catalysis [4], and others [5,6,7]. The range of applications for porphyrins can be attributed to some features observed in these molecules, including invariable planar shapes, effective absorption and emission, and high stability under different hazardous conditions [8,9,10,11]. Despite the numerous applications for porphyrins, their use in biomedical devices can be highlighted as one of their most prominent features. Porphyrins show photoactivity in some neoplastic diseases [12,13,14,15,16,17] and can be used as antimicrobial agents [18,19,20,21]. The most notable behavior observed in porphyrins to achieve these applications is their ability to generate singlet oxygen species (^1^O_2_) [22]. This process initially involves porphyrin energy absorption and formation of a triplet-excited state. Following this, a transfer of energy from the excited porphyrin to molecular oxygen affords the cytotoxic ^1^O_2_ species [23,24,25,26,27,28]. In this context, modulating physicochemical parameters in porphyrins to make them more tunable for specific applications is an exciting and promising field of study. In this context, thioaryl-porphyrins derived from 5,10,15,20-tetrakis(pentafluorophenyl)porphyrin (TPPF_20_) are a class of extensively documented porphyrins that have sulfur atoms in their structures. Generally, they are obtained via thiols with high yields and require either long reaction periods, high temperatures or react only with porphyrin complexed with certain metals, such as Zn(II) and Mn(III) [10,29,30,31]. 

**Scheme 1:**
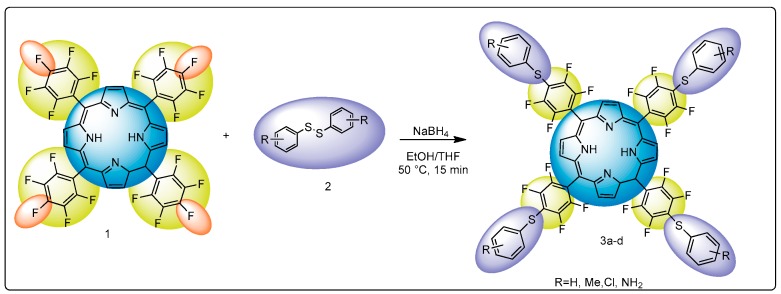
Synthesis of thioaryl-porphyrins **3a–d.**

Based on this, this study aims to describe a new methodology to prepare thioaryl-porphyrins via a reductive cleavage of disulfides in the presence of TPPF_20_. For this new class of thioaryl-porphyrins, the photophysical parameters were evaluated as well as the ct-DNA interactions by spectroscopic methods (Scheme 1).

## 2. Results and Discussion

Diphenyl disulfide and TPPF_20_ were used as standard to prepare compound **3a** and to optimize the reactional conditions. Initially, TPPF_20_ (10 µmol; 10 mg), diphenyl disulfide (80 µmol; 17.4 mg), THF (7 mL), EtOH (3 mL), and NaBH_4_ (180 µmol; 6.8 mg) were added to a reaction flask. The system was stirred at 50 °C and the reaction monitored by thin layer chromatography (TLC) for 15 min. In this case, the respective thioaryl-porphyrin **3a** was obtained in 84% yield. In order to find the best reactional condition, disulfide variation was evaluated, while keeping the reaction times. When we reduced the amount of diphenyl disulfide to 40 µmol (8.7 mg), the respective compound **3a** was obtained in 70% yield. Additionally, the effect of time was studied. In this case, when the reaction was carried out in one hour, it afforded thioaryl-porphyrin **3a** in 77% yield. Moreover, decreasing the reaction time to five minutes allowed the preparation of compound **3a** in 53% yield. After optimization, the best reaction was achieved by using 80 µmol of diphenyl disulfide for 15 min at 50 °C. 

With the optimal reactional condition established, the versatility of the protocol was explored using different diaryldisulfides, containing either activating or deactivating groups attached to the aromatic ring (Table 1).

The compounds were obtained in very-good to excellent yields for all disulfides used (Table 1). Despite this, the activating groups attached to the aromatic ring in the disulfide portion afforded better yields compared to the deactivating ones. For instance, the activating methyl and amine groups in compounds **3b** and **3d** (Table 1, Entries **2** and **4**) afforded thioaryl-porphyrins with 92 and 93% yields, respectively. The presence of a chlorine group attached to the aromatic ring decreased the yield to 82% (Entry **3**). This behavior may be explained by the higher nucleophilicity of the thiolates with activating, instead of deactivating, groups attached to the aromatic ring in the disulfides moieties.

### 2.1. General Absorption and Emission Properties of Thioaryl-Porphyrins ***3a**–**d***

**Figure 1:**
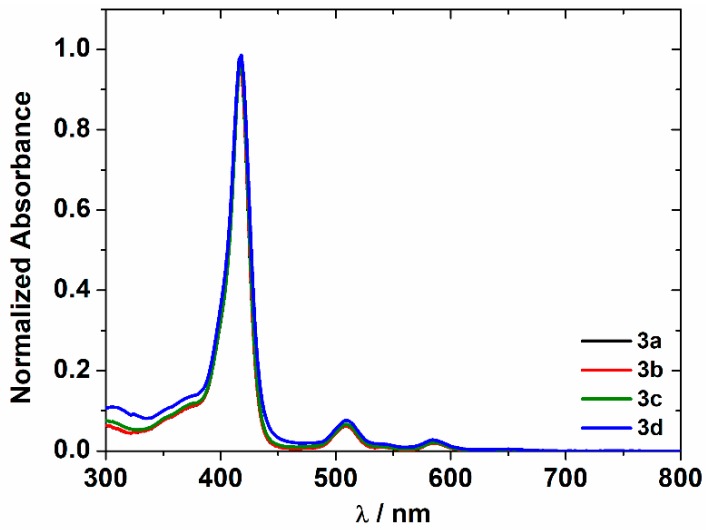
Normalized UV-vis absorption spectra of thioaryl-porphyrins **3a**–**d** containing different substituent groups in CHCl_3_ solutions.

Porphyrins **3a**–**d** in chloroform solution show a Soret band transition (π → π* macrocycle ring) at about 417 nm and four vibronic Q band transitions at 508–652 nm (Figure 1). All porphyrins had about the same molar absorptivity (log ε; Table 2), which is in agreement with the molecular structures. Moreover, it seems that the aryl-sulfur electron-withdrawing or -donating moiety does not change the band position or peak magnitude, and therefore, it is very likely that it was overlapped by the Soret band intensity.

**Figure 2:**
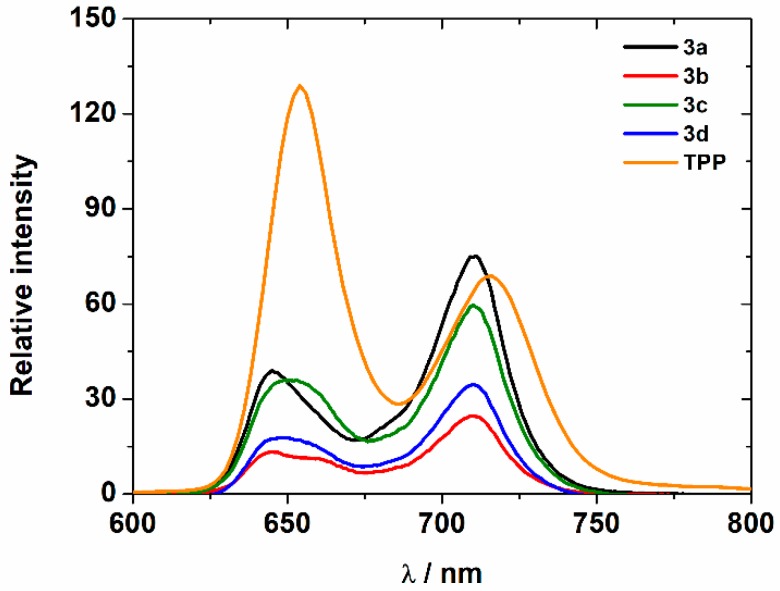
Emission spectra of porphyrin derivatives (excitation at λ_exc_ = 418 nm) in argon saturated CHCl_3_ solutions.

The emission spectra analysis of thioaryl-porphyrins **3a**–**d** dissolved in dry chloroform solution (λ_exc_ = 418 nm) are shown in Figure 2 and Table 2. The fluorescence emission quantum yields (Φ_f_) for sulfur-derivatives were estimated from the reference 5,10,15,20-tetraphenylporphyrin (TPP) by comparative method. The fluorescence quantum yield of molecules indicates the capacity of an excited compound (in the first excited state) to return to the electronic ground state by photon emission. This process depends on the electronic molecular structure, solvent interaction type, and stereochemistry. The values of fluorescence quantum yields were determined at an optical density (OD) in the range 0.01–0.03. By inserting the aryl-sulfur units with different electronic groups at the *para*-position of the *meso*-aryl moieties, the fluorescence quantum yield decreased when compared to the TPP standard. This may be explained by the presence of the halogen atoms in the *meso*-aryl moieties [32]. Moreover, the spin-orbit coupling factor of the halogen atoms decreased radiative channels and increased non-radiative channel processes [32,33,34]. 

### 2.2. Singlet Oxygen Generation (^1^O_2_) Experiments

**Figure 3:**
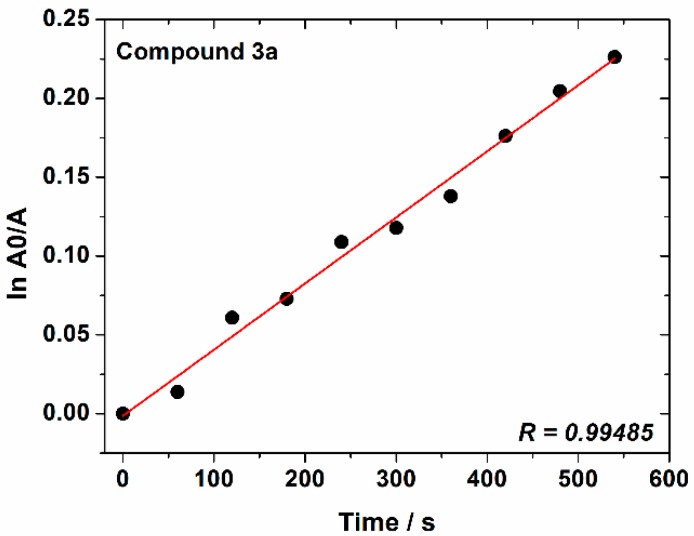
First-order kinetic graphical profile of photodegradation of 1,3-diphenylisobenzofuran (DPBF) by irradiation with LED array system (λ = 635 nm) in the presence of thioaryl-porphyrin **3a**.

The ability of porphyrins **3a**–**d** to produce ^1^O_2_ was monitored using 1,3-diphenylisobenzofuran (DPBF) in aprotic solvent (DMF) [36]. The DPBF photo-oxidation method has been widely used to quantitatively analyze singlet oxygen production because the reaction product (1,2-dibenzoylbenzene) does not absorb in the visible region. In this assay, changes in DPBF absorbance are directly related to the amount of ^1^O_2_ generated [37]. This can be observed in Figure 3, in which the first-order kinetic profile of DPBF photo-oxidation in the presence of thioaryl-porphyrin **3a** was monitored at 415 nm during irradiation with a red-light LED array system (λ = 635 nm) in DMF solution. One of the important parameters in which to evaluate the photodynamic potential of a photosensitizer is the production of singlet oxygen, (Φ_Δ_), which is one of the most important reactive oxygen species (ROS) in photophysical and photochemical processes [38]. In this study, the singlet oxygen quantum yield of porphyrins **3a**–**d** was determined, and the values are presented in Table 3. As observed in Table 3, the Φ_Δ_ found for compound **3a** is close to the standard TPP and porphyrin **1** in several solvents [39,40,41]. In the case of porphyrins **3b**–**d**, which have S-aryl units with different substituents (**b**:4-CH_3_, **c**:4-Cl, **d**:2-NH_2_), the observed values are smaller than TPP ($\Phi_{\Delta }^{\mathrm{std}}$ = 0.66) [38,42], although they still displayed an ability to generate singlet oxygen. This behavior could be attributed to electronic deactivation pathways of the excited state due to the presence of the substituents on the thioaryl moieties. Although the ability of sulfur-porphyrin derivatives to generate ^1^O_2_ is, in general, lower than the TPP or the starting porphyrin **1**, all the thioaryl derivatives, after being exposed to red light in the presence of oxygen, demonstrate potential for photodynamic therapy (PDT) applications.

### 2.3. Biomolecule Interactive Studies

#### 2.3.1. DNA-Binding Assays by Absorption and Emission Analysis

**Figure 4:**
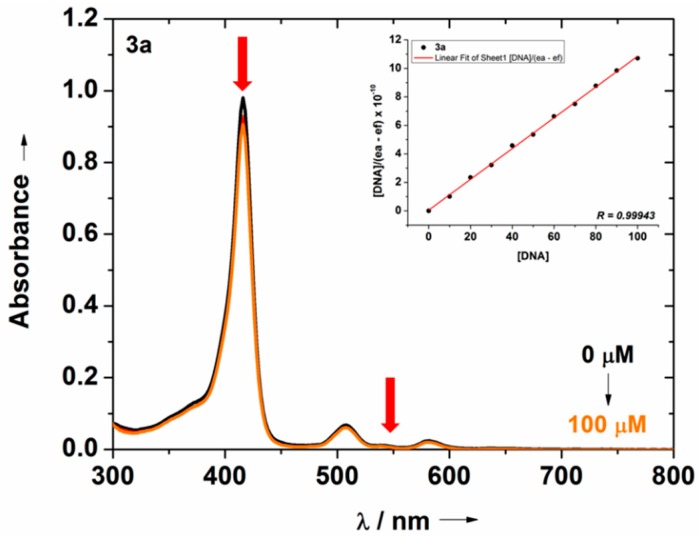
UV-vis titration absorption spectra of thioaryl-porphyrin **3a** in a DMSO/Tris-HCl buffer (pH 7.4) mixture. The ct-DNA concentration ranged from 0 to 100 μM. The insert graph shows the plot of [DNA]/(ε_a_ − ε_f_) versus [DNA].

The interaction of thioaryl-porphyrins **3a**–**d** with ct-DNA has also been studied by UV-vis absorption spectroscopy at the 300–800 nm range in DMSO (1%)/Tris-HCl buffer solution mixture at pH 7.4. Thioaryl-porphyrin derivatives interact with DNA and decrease the transition band at a visible range (Soret band). The effect of different ct-DNA concentrations on the Soret and Q-band transitions in the absorption spectrum of compound **3a** (as an example) is presented in Figure 4.

In general, upon interaction with the ct-DNA concentration, porphyrin derivatives **3a**–**d** revealed distinctive changes (slight hypochromicity; *H*%) in the UV-vis absorption electronic spectra (Table 4). The addition of various ct-DNA concentrations (0–100 μM) decreased Soret band intensity. No red shift was observed in any case, which may indicate the non-observed electrostatic interaction of the porphyrin molecules and nucleic acids (Figure 4; Table 4). Furthermore, the changes in intensity of the π→π* Soret transition may be due to the interaction of the aromatic structure of the tetrapyrrolic derivatives, peripheral groups, or both, which is likely because of H-bonding interactions with the DNA nucleobases. This effect is probably caused by the presence of the π-density macrocycle moiety in the structure that may interact with DNA via hydrophobic interactions, as previously reported for some cases of porphyrin derivatives, and possibly by H-bonding interactions [32,43].

The constant binding values (K_b_) of the thioaryl-porphyrins were calculated using Equation (2) (see ESI) (Table 4). In this study, porphyrin derivatives containing S-aryl units demonstrated stronger binding to ct-DNA (K_b_ ~10^6^ M^−1^), following the increasing order of K_b_ data: **3c** < **3b** < **3a** < **3d**. All DNA UV-vis titration spectra of thioaryl-porphyrins **3b**–**d** are listed in the supporting information (Figures S9–S11).

**Figure 5:**
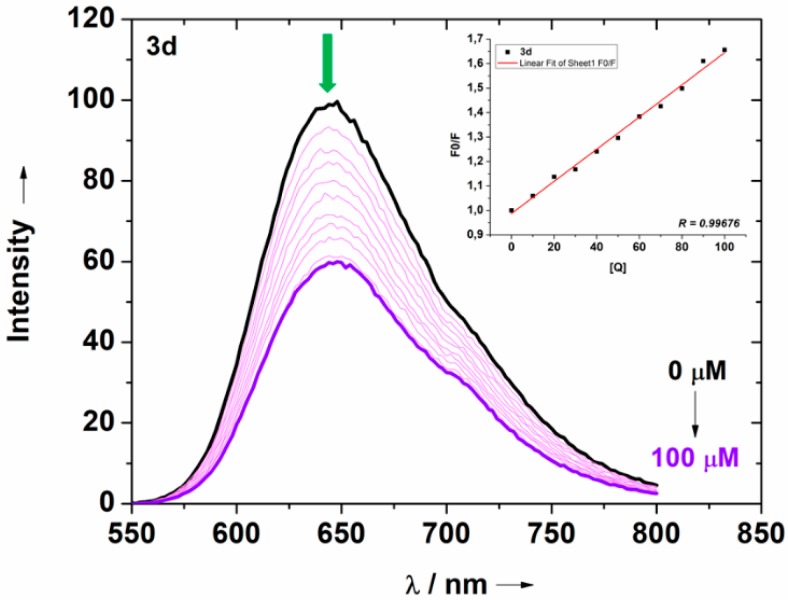
Emission fluorescence spectra of ethidium bromide (EB) bound to ct-DNA in the presence of porphyrin **3d** in a DMSO/Tris-HCl buffer mixture at λ_exc_ = 510 nm. The arrow indicates the changes in fluorescence intensities at increasing sample concentrations. The insert graph shows the plot of F_0_/F versus [ct-DNA].

Competitive-binding assays using the well-known quenching fluorescence method experiment to determine the displacement of the intercalating ethidium bromide (EB) from ct-DNA may provide further confirmation of the binding affinity of the compounds. Emission fluorescence spectra were monitored as increasing concentrations of porphyrins were added by titration to a fixed ct-DA concentration pre-treated with EB (Figure 5).

When thioaryl-porphyrin derivatives were added to the DNA pre-treated with EB, the DNA-induced emission intensity of EB decreased (Figure 5). The EB-DNA interaction with porphyrin **3d** showed strong emission at λ_em_ = 644 nm, and, when excited, at λ_exc_ = 510 nm, in addition to also depicting the emission spectra of EB bound to ct-DNA in both the absence and presence of the porphyrin.

These results demonstrate a quenching of the fluorescence intensity of the EB-DNA adduct following the addition of increasing concentrations of porphyrin compounds (Figure 5). This spectral behavior may be attributed to the competition of the porphyrin moiety with the EB intercalator over binding to the DNA structure. In this context, the K_SV_ values suggest the competition mode of EB-binding. Moreover, the higher values observed for the quenching constant rate (*k*_q_) indicated a static interaction between the porphyrins and DNA (Table 4).

The inset graphs in Figure 5 show the Stern-Volmer plot, which was obtained as the relationship between F_0_/F and ct-DNA concentrations. The quenching Stern-Volmer constant (K_SV_) and quenching constant rate (*k*_q_) values are presented in Table 4. Moreover, EB-DNA emission spectra of derivatives **3a**–**c** are listed in the supporting information section (Figures S12–S14).

#### 2.3.2. DNA Molecular Docking with Thioaryl-Porphyrins **3a**–**d**

**Figure 6:**
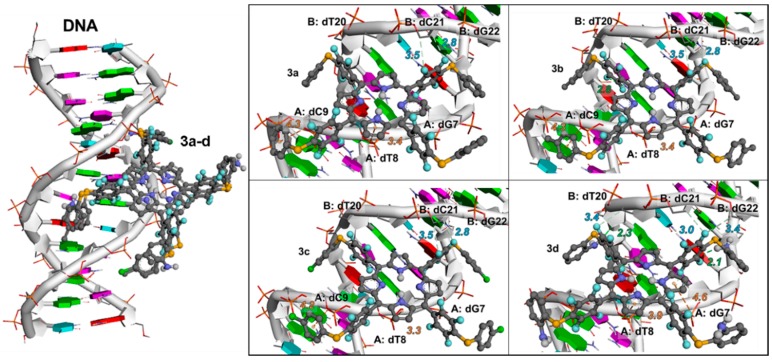
Overview of the interactions between DNA and compounds **3a**–**d**. Both compounds interact in the minor groove region of the DNA. The dotted lines represent the respective interactions: green indicates H-bonds; orange represents the π-anion interactions; light blue indicates carbon-hydrogen bonds. Rings in red, blue, pink, and green (in DNA) indicate deoxyadenosine (dG), deoxythymidine (dT), deoxycytidine (dC), and deoxyguanosine (dG), respectively. The distances of the interactions are in Å.

A molecular docking simulation was carried out in order to better understand the interactions of compounds **3a**–**d** with DNA. The molecular docking with DNA showed that thioaryl-porphyrin derivatives interact in the minor groove region with a very similar binding pose (Figure 6). All molecules bind in the region between the nucleoside residues deoxyadenosine-7 (dG-7), deoxythymidine-8 (dT-8), deoxycytidine-9 (dC-9) from the DNA chain A, deoxyadenosine-20 (dG-20), deoxycytidine-21 (dC-21) and deoxyguanosine-22 (dG-22) from DNA chain B (Figure 6, left side). In fact, some studies have even indicated that thioaryl-porphyrin hybrids can bind to the minor groove of DNA [44,45]. 

In general, the compounds showed electrostatic π-anion interactions between the phosphate groups (from DNA) and the pyrrolic and aryl ring from compounds **3a**–**d**, in addition to H-bonds between the fluorine group and an amino moiety from the DNA bases. These observations indicate that the substituent groups do not significantly interfere in the interactions, despite compound **3d** (which possesses the *ortho*-amino group) presenting more H-bonds with the DNA. In addition, we observed the importance of the carbon-hydrogen bond with the fluorine moiety (C-H···F-C) [46,47,48,49] as an interaction that stabilizes the thioaryl-porphyrins-DNA adduct. The predicted theoretical thermodynamic data (∆G_bind_) indicated that all the thioaryl-porphyrin derivatives present spontaneous interaction with DNA fragments (**3a** (−8.8 kcal/mol); **3b** (−9.3 kcal/mol); **3c** (−9.3 kcal/mol); **3d** (−8.8 kcal/mol)). This is probably due to the steric hindrance of the thioaryl-porphyrin derivatives since they bind to the minor groove of DNA and do not intercalate [49].

## 3. Materials and Methods 

Chemistry: Hydrogen nuclear magnetic resonance (^1^H-NMR) spectra were obtained on a Bruker Avance III spectrometer, that operate on the frequency of 600 MHZ to hydrogen, at the Universidade Federal de Santa Maria. Spectra were recorded in CDCl3 solutions. Chemical shifts are reported in parts per million, referenced to the solvent peak of TMS. Data are reported as follows: chemical shift (d), multiplicity (s = singlet, d = doublet, dd = double doublet, t = triplet, m = multiplet), and coupling constant (J) in hertz and integrated intensity. Fluor-19 nuclear magnetic resonance (^19^F) spectra were obtained at 565 MHz. Spectra were recorded in CDCl3 solutions and C6F6 as external reference. High resolution mass spectra: Samples were diluted in methanol, containing 100 µL of 200 mM NH4OH. Analyses were performed by infusion mode in an ACQUITYTMUPLC system from Waters Corp. (Milford, MA, USA) equipped with sampler manager and quadrupole time of flight (Q-Tof) MS detector. The Q-Tof Xevo G2 mass spectrometer was equipped with an electrospray ionization source (ESI). Detections were performed in positive ion mode (ESI+) and resolution mode. Optimized MS conditions were: capillary voltage 2.50 kV, cone voltage 112 V, extractor cone 4.5 V, desolvation gas 500 L/h, cone gas 10 L/h, desolvation temperature 400 °C, and source temperature 150 °C. Acquisition mass range was monitored from 50 to 1800 Da. System control and data acquisition were performed using MassLynx V 4.1 software.

### General Procedure for the Synthesis ***3a**–**d***

In a Schlenk tube under an argon atmosphere, 0.08 mmol of the respective diaryldisulfide, THF (7 mL), sodium borohydride (180 µmol; 6.8 mg), and ethanol (3 mL) were added. After 1 min, TPP-F20 (10 µmol; 10 mg) 1 was added, and the resulting mixture was stirred at 50 °C for 15 min. The reaction was quenched with 10 mL of water, and the aqueous layer was extracted with CH_2_Cl_2_. The combined organic extracts were dried over MgSO_4_, filtered and evaporated to dryness. The crude products were purified in a silica gel TLC for chromatographic purification, using hexane–ethylacetate (70:20) as the eluent, affording the pure porphyrin **3a**–**d**.

*5,10,15,20-tetrakis[4-(phenylthio)-2,3,5,6-tetrafluorophenyl]porphyrin* (**3a**)*.* Physical state: purple solid. Yield: 10.9 mg, 84%. ^1^H-NMR (600 MHz, CDCl3) δ 8.91 (s, 8H), 7.70 (d, *J* = 6 Hz, 8H), 7.41–7.49 (m, 12H), −2.85 (s, 2H) ppm. ^19^F-NMR (565 MHz, CDCl3) δ −132.87 (dd, *J*_1_ = 22.6, *J*_2_ = 11.3 Hz, F *ortho*), −136.29 (dd, *J*_1_ = 22.6, *J*_2_ = 11.3 Hz, F *meta*) ppm. HRMS-ESI: *m*/*z* calcd to to C_68_H_30_F_16_N_4_S_4_ [M + H]^+^; 1335.1176 found:1335.1223.

*5,10,15,20-tetrakis[-(4-methylphenylthio)-2,3,5,6-tetrafluorophenyl]porphyrin* (**3b**). Physical state: purple solid. Yield: 12 mg, 92%. ^1^H-NMR (600 MHz, CDCl3) δ 8.90 (s, 8H), 7.65 (d, *J* = 6 Hz, 8H), 7.29 (d, *J* = 6 Hz, 8H), 2.44 (s, 12H), −2.91 (s, 2H). ^19^F-NMR (565 MHz, CDCl3) δ −133.28 (dd, *J*_1_ = 28.2, *J*_2_ = 11.3 Hz, F *ortho*), −136.49 (dd, *J*_1_ = 28.2, *J*_2_ = 11.3 Hz, F *meta*) ppm. HRMS-ESI: *m*/*z* calcd to C_72_H_38_F_16_N_4_S_4_ [M + H]^+^; 1391.1802 found: 1391.1873.

*5,10,15,20-tetrakis[4-(4-chlorophenylthio)-2,3,5,6-tetrafluorophenyl]porphyrin* (**3c**). Physical state: purple solid. Yield: 10.7 mg, 82%. ^1^H-NMR (600 MHz, CDCl3) δ 8.90 (s, 8H), 7.66 (d, *J* = 6 Hz, 8H), 7.47 (d, *J* = 6 Hz, 8H), −2.89 (s, 1H). ^19^F-NMR (565 MHz, CDCl3) δ −132.80 (dd, *J*_1_ = 22.6, *J*_2_ = 11.3 Hz, F *ortho*), −135.90 (dd, *J*_1_ = 22.6, *J*_2_ = 11.3 Hz, F *meta*) ppm. HRMS-ESI: *m*/*z* calcd to C_68_H_26_Cl_4_F_16_N_4_S_4_ [M + H]^+^; 1472.9588 found: 1472.9628.

*5,10,15,20-tetrakis[4-(2-aminophenylthio)-2,3,5,6-tetrafluorophenyl]porphyrin* (**3d**). Physical state: purple solid. Yield: 12.9 mg, 93%. ^1^H-NMR (600 MHz, CDCl3) δ 8.86 (s, 8H), 7.83–7.81 (m, 4H), 7.32–7.29 (m, 4H), 6.88–6.83 (m, 8H), 4.68 (s, 8H) −2.96 (s, 2H). ^19^F-NMR (565 MHz, CDCl3) δ −133.78 (dd, *J*_1_ = 28.2, *J*_2_ = 11.3 Hz, F *ortho*), −136.53 (dd, *J*_1_ = 28.2, J_2_ = 11.3 Hz, F *meta*) ppm. HRMS-ESI: *m*/*z* calcd to C68H34F16N8S4 [M + H]^+^; 1395.1612 found: 1395.1611.

## 4. Conclusions

The synthesis of thioaryl-porphyrins using a new synthetic protocol was disclosed. The methodology enabled the preparation of four porphyrins in very-good to excellent yields under soft conditions. The new compounds presented good photophysical properties to be considered as photosensitizers for photodynamic therapy. Additionally, experimental and theoretical DNA interactive assays were performed, showing an effective interaction between thioaryl-porphyrin hybrids and DNA.

## Figures and Tables

**Table 1:** Preparation of thioaryl-porphyrin **3a**–**d**.

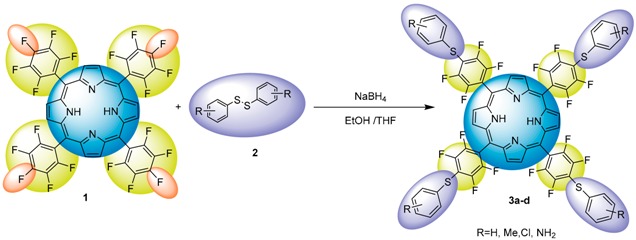		
**Entry**	**Product**	**Yield (%) ^a^**
1	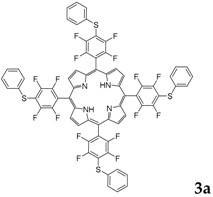	84
2	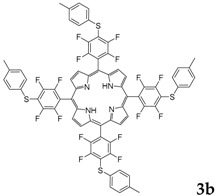	92
3	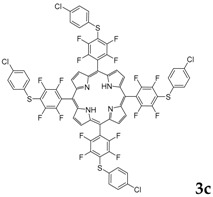	82
4	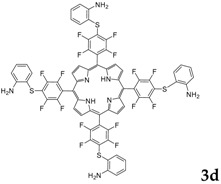	93

^a^ Yields are related to isolated and purified products.

**Table 2:** Absorption and emission data of porphyrin derivatives **3a**–**d**.

Porphyrin	nm, λ (log ε; M^−1^cm^−1^) ^a^	Emission (nm) ^b^	Φ_f_^c^
**3a**	417 (5.00), 509 (3.81), 542 (3.00), 585 (3.30), 648 (2.60)	648, 710	0.03
**3b**	417 (5.04), 509 (3.85), 540 (3.04), 585 (3.34), 647 (2.65)	648, 710	0.01
**3c**	418 (5.02), 508 (3.86), 542 (3.08), 585 (3.36), 652 (2.62)	652, 710	0.02
**3d**	418 (5.07), 509 (3.96), 543 (3.30), 584 (3.52), 646 (2.69)	649, 710	0.01

^a^ Measured in chloroform ([ ] = 10^−5^ M range); ^b^ Measured in dry chloroform solution ([ ] = 2.0 × 10^−7^ M) at 298 K (λ_exc_ = 418 nm); ^c^ TPP in DCM as standard (Φ_f_ = 0.15) [35].

**Table 3:** Singlet oxygen quantum yields of thioaryl-porphyrins **3a–d**.

Porphyrin	Φ_Δ_
**3a**	0.67
**3b**	0.10
**3c**	0.11
**3d**	0.14
TPP *	0.66
**1**	0.70 ^a^ [39]; 0.80 ^b^ [40], 0.60 ^c^ [41]

* As standard in DMF solution.; ^a^ in benzene. ^b^ in dichloromethane; ^c^ in toluene.

**Table 4:** DNA-binding data of thioaryl-porphyrin derivatives **3a**–**d**.

Porphyrin	Absorption			Emission		
	*H*(%) ^a^	Δλ (nm) ^b^	K_b_ (M^−1^) ^c^	*Q*(%) ^d^	K_SV_ (M^−1^) ^e^	*k*_q_ (s^−1^M^−1^) ^f^
**3a**	7.65	0.0	2.08 × 10^6^	32.58	4.92 × 10^3^	2.14 × 10^12^
**3b**	4.72	0.0	1.27 × 10^6^	3.60	3.30 × 10^2^	1.43 × 10^11^
**3c**	3.22	0.0	0.76 × 10^6^	21.28	2.66 × 10^2^	1.15 × 10^12^
**3d**	6.31	0.0	2.86 × 10^6^	39.56	6.57 × 10^3^	2.85 × 10^12^

^a^*H*(%) = (Abs_initial_ − Abs_final_)/(Abs_initial_) × 100 (at Soret band); ^b^ Δλ(nm) = λ_final_ – λ_initial_ (at Soret band); ^c^ Binding constant by UV-vis ct-DNA analysis; ^d^ *Q*(%) = (Emission_initial_ − Emission_final_)/(Emission_initial_) × 100; ^e^ Stern-Volmer quenching ethidium bromide-DNA (EB-DNA) constant by emission spectra; ^f^ Stern-Volmer rate quenching EB-DNA constant by emission analysis.

## Supplementary Materials

The following are available online. Experimental procedures, characterization of compounds, DNA UV-vis absorption spectra, EB DNA emission fluorescence spectra.
