# Supplementary material for: A New Protocol for the Synthesis of New Thioaryl-Porphyrins Derived from 5,10,15,20-Tetrakis(pentafluorophenyl)porphyrin: Photophysical Evaluation and DNA-Binding Interactive Studies
Source: Molecules. 2018 Oct 10;23(10):2588. doi: 10.3390/molecules23102588 (PMC6222446; doi:10.3390/molecules23102588)
Supplement: Supplementary file 1 [file molecules-23-02588-s001.pdf]

Electronic Supplementary Information (ESI)

Patrícia Foletto[1], Fabiula Correa[1], Luciano Dornelles[1], Bernardo A. Iglesias[2,*], Carolina H. da Silveira[2], Pablo A. Nogara,[3] João B. T. da Rocha,[3*] Maria A. F. Faustino[4] and Oscar E. D. Rodrigues[1,*]

[1] Universidade Federal de Santa Maria, Departamento de Quimica, Santa Maria, RS, 97105-900, Brazil;

[2] Departamento de Química, Laboratório de Bioinorgânica e Materiais Porfirínicos, Universidade Federal de Santa Maria – UFSM, ZIP CODE 97105-900, Santa Maria – RS, Brazil;

[3]Laboratório de Bioquímica Toxicológica, Departamento de Bioquímica e Biologia Molecular, Univerisidade Federal de Santa Maria, RS, Brazil.

[4] QOPNA and Department of Chemistry, University of Aveiro, 3810-193, Aveiro, Portugal

[a] Universidade Federal de Santa Maria, Santa Maria, RS, 97105-900, Brazil, Fax: :+55 55 3220 8761

e-mail: rodriguesoed@smail.ufsm.br, bernardopgq@gmail.com

TABLE OF CONTENTS

1. **General Information…………………………………………………………… 03**
2. **General procedure for the synthesis 3 a-d……………………..……………... 06**
3. **Compounds Description: NMR………………………………………..………… 08**
4. **High resolution mass spectra……………………………………………..……… 12**
5. **DNA UV-vis absorption spectra………………………………………….……… 14**
6. **EB-DNA emission fluorescence spectra…………………………………..…... 15**
7. **Porphyrin docking with DNA 17**

References... 18

1. General Information

Chemistry: Hydrogen nuclear magnetic resonance (^1H NMR) spectra were obtained on a Bruker Avance III spectrometer, that operate on the frequency of 600 MHZ to hydrogen, at the Universidade Federal de Santa Maria. Spectra were recorded in $CDCl_3$ solutions. Chemical shifts are reported in parts per million, referenced to the solvent peak of TMS. Data are reported as follows: chemical shift (d), multiplicity (s = singlet, d = doublet, dd = double doublet, t = triplet, m = multiplet), and coupling constant (*J*) in hertz and integrated intensity. Fluor-19 nuclear magnetic resonance (^{19}F) spectra were obtained at 565 MHz. Spectra were recorded in $CDCl_3$ solutions and C_6F_6 as external reference.

High resolution mass spectra

Samples were diluted in methanol, containing 100 µL of 200 mM NH_4OH. Analyses were performed by infusion mode in an ACQUITY™UPLC system from Waters Corp. (Milford, MA, USA) equipped with sampler manager and quadrupole time of flight (Q-Tof) MS detector. The Q-Tof Xevo G2 mass spectrometer was equipped with an electrospray ionization source (ESI). Detections were performed in positive ion mode (ESI+) and resolution mode. Optimized MS conditions were: capillary voltage 2.50 kV, cone voltage 112 V, extractor cone 4.5 V, desolvation gas 500 L/h, cone gas 10 L/h, desolvation temperature 400 °C, and source temperature 150 °C. Acquisition mass range was monitored from 50 to 1800 Da. System control and data acquisition were performed using MassLynx V 4.1 software.

UV-vis absorption spectra were recorded using Shimadzu UV2600 spectrophotometer (data interval, 1.0 nm) using chloroform as solvent. Steady state emission fluorescence spectra of samples in $CHCl_3$ solutions were measured with a Varian Cary 50 fluorescence spectrophotometer (slit 2.0 mm) and were corrected according to the manufacturer's instructions. Fluorescence quantum yields (Φ_f) of the compounds in solutions were determined by comparing the corrected fluorescence spectra with that of *meso*-tetra(phenyl)porphyrin in dichloromethane (TPP, $\Phi_f = 0.15$, $\lambda_{exc} = 418$ nm) as the standard as the fluorescence yield [1]

Singlet oxygen generation ($^1O_2$) assays

In a typical experiment of 1.3-diphenylisobenzofuran (DPBF) photo-oxidation [2], 2.0 mL of 100 µM DPBF in DMF was mixed with 0.5 mL (50 µM) of porphyrins. The flask

was completed with DMF until a final volume of 3.0 mL. In order to measure $^1O_2$ generation species, absorption spectra of the solutions (samples and standard) were recorded for different exposure times by using a 635 nm home-made LED array system positioned 1.0 cm from the sample, with an average power of 50 mW[3].

$$\Phi_\Delta = \Phi_\Delta^{\mathrm{std}} \, {k}/{k^{\mathrm{std}}} \cdot {I^{std}}/{I} \qquad (1)$$

in which, $\mathrm{I^{std}/I} = (1 - 10^{A_{std}})/(1 - 10^{A})$, $\Phi_\Delta^{\mathrm{std}}$ is the singlet oxygen quantum yield of standard sample (in our case, *meso*-tetra(phenyl)porphyrin; TPP; $\Phi_\Delta^{\mathrm{std}} = 0.66$) [4], k and k^{std} are the photodegradation kinetic constants for porphyrins and TPP (standard), respectively, and A_{std} and A are the absorbances for TPP and porphyrins, respectively.

Absorption and emission analysis with ct-DNA

For acid nucleic assays, sulfur-porphyrin compounds interactions with calf-thymus DNA (ct-DNA) were performed by UV-vis absorption measurements at room temperature in Tris-HCl buffer at pH 7.4, using DMSO stock solution of derivatives (10^{-4} M range) at 300 to 700 nm. The DNA pair base concentrations of low molecular weight DNA from calf thymus (ct-DNA) was determined by absorption spectroscopy, using the molar extinction coefficients 6600 $M^{-1}cm^{-1}$ (per base pair) at 260 nm, respectively. Derivative solutions in DMSO with Tris-HCl buffer were titrated with increasing concentrations of DNA (ranging from 0-100 μM). The absorption spectra of derivatives were acquired in the wavelength range of 300–700 nm. The intrinsic binding constants (K_b) of derivatives were calculated according to the decay of the absorption bands of compounds using the following Equation (2) through a plot of [DNA]/($\varepsilon_a - \varepsilon_f$) *versus* [DNA],

$$[\mathrm{DNA}]/(\varepsilon_a - \varepsilon_f) = [\mathrm{DNA}]/(\varepsilon_b - \varepsilon_f) + 1/K_b(\varepsilon_b - \varepsilon_f) \qquad (2)$$

where [DNA] is the concentration of DNA in the base pairs, ε_a is the extinction coefficient (A_{obs}/[compound]), ε_b and ε_f are the extinction coefficients of free and fully bound forms, respectively. In plots of [DNA]/($\varepsilon_a - \varepsilon_f$) *versus* [DNA], K_b is given by the ratio of the slope to the interception.

In competition EB-DNA assays, steady-state emission fluorescence analysis was

recorded and porphyrin derivatives were dissolved in DMSO and competitive studies were performed through the gradual addition of the stock solution of the derivatives to the quartz cuvette (1.0 cm path length) containing ethidium bromide (EB, 2.0 x 10^{-7} M) and DNA (2.0 x 10^{-5} M) in a Tris-HCl pH 7.4 buffer solution. The concentration of derivatives ranged from 0 to 100 μM. Samples were excited at λ_{exc} = 510 nm and emission spectra were recorded at the range of 550-800 nm, 5 min after each addition of the complex solution in order to allow incubation to occur. The fluorescence quenching Stern-Volmer constants (K_{SV}) of compounds were calculated according to the decay of the emission bands of EB-DNA using the following Equation (3) through a plot of F_0/F *versus* [DNA],

$$F_0/F = 1 + k_q\tau_0[Q] = 1 + K_{SV}[Q] \qquad (3)$$

where F and F_0 are the fluorescence intensities in the presence and absence of a quencher, respectively. K_{SV}, k_q, τ_0 and [Q] denote Stern–Volmer quenching constant, quenching rate constant, lifetime of EB-DNA adducts (2.30 x 10^{-9} s) and the concentration of quencher, respectively. According to Equation (3), the Stern–Volmer constants (K_{SV}) were calculated from the slope and k_q is equal K_{SV}/τ_0.

2. General procedure for the synthesis 3 a-d

In a Schlenk tube under an argon atmosphere, 0.08 mmol of the respective diaryldisulfide, THF (7 mL), sodium borohydride (180 **µmol**; 6.8 mg), and ethanol (3 mL) were added. After 1 min, TPP-F_{20} (10 **µmol**; 10 mg) **1** was added, and the resulting mixture was stirred at 50 °C for 15 min. The reaction was quenched with 10 mL of water, and the aqueous layer was extracted with CH_2Cl_2. The combined organic extracts were dried over $MgSO_4$, filtered and evaporated to dryness. The crude products were purified in a silica gel TLC for chromatographic purification, using hexane–ethylacetate (70:20) as the eluent, affording the pure porphyrin **3 a-d**.

5,10,15,20-tetrakis[4-(phenylthio)-2,3,5,6-tetrafluorophenyl]porphyrin (3a)

Physical state: purple solid. Yield: 10.9 mg, 84%.

^{1}H NMR (600 MHz, $CDCl_3$) δ 8.91 (s, 8H), 7.70 (d, *J* = 6 Hz, 8H), 7.41-7.49 (m, 12H), -2.85 (s, 2H) ppm.

^{19}F NMR (565 MHz, $CDCl_3$) δ -132.87 (dd, J^1 = 22.6, J^2=11.3 Hz, F *ortho*), -136.29 (dd, J^1= 22.6, J^2=11.3 Hz, F *meta*) ppm.

HRMS-ESI: m/z calcd to to $C_{68}H_{30}F_{16}N_4S_4$ $[M+H]^+$; 1335.1176 found:1335.1223.

5,10,15,20-tetrakis[4-(4-methylphenylthio)-2,3,5,6-tetrafluorophenyl]porphyrin (3b) Physical state: purple solid. Yield: 12 mg, 92%.

^{1}H NMR (600 MHz,) δ 8.90 (s, 8H), 7.65 (d, *J* = 6 Hz, 8H), 7.29 (d, *J* = 6 Hz, 8H), 2.44 (s, 12H), -2.91 (s, 2H).

^{19}F NMR (565 MHz,) δ -133.28 (dd, J^1 = 28.2, J^2=11.3 Hz, F *ortho*), -136.49 (dd, *J* = 28.2, J^2=11.3 Hz, F *meta*) ppm.

HRMS-ESI: m/z calcd to $C_{72}H_{38}F_{16}N_4S_4$ $[M+H]^+$; 1391.1802 found: 1391.1873

5,10,15,20-tetrakis[4-(4-chlorophenylthio)-2,3,5,6-tetrafluorophenyl]porphyrin (3c)

Physical state: purple solid. Yield: 10.7 mg, 82%.

^{1}H NMR (600 MHz, $CDCl_3$) δ 8.90 (s, 8H), 7.66 (d, *J* = 6 Hz, 8H), 7.47 (d, *J* = 6 Hz, 8H), -2.89 (s, 1H).

^{19}F NMR (565 MHz, $CDCl_3$) δ -132.80 (dd, J^1 = 22.6, J^2=11.3 Hz, F *ortho*), -135.90 (dd, J^1 = 22.6, J^2=11.3 Hz, F *meta*) ppm.

HRMS-ESI: m/z calcd to $C_{68}H_{26}Cl_4F_{16}N_4S_4$ $[M+H]^+$; 1472.9588 found:. 1472.9628

5,10,15,20-tetrakis[4-(2-aminophenylthio)-2,3,5,6-tetrafluorophenyl]porphyrin (3d)

Physical state: purple solid. Yield: 12.9 mg, 93%.

^{1}H NMR (600 MHz, $CDCl_3$) δ 8.86 (s, 8H), 7.83-7.81 (m, 4H), 7.32-7.29 (m, 4H), 6.88-6.83 (m, 8H), 4.68 (s, 8H) -2.96 (s, 2H).

^{19}F NMR (565 MHz, $CDCl_3$) δ -133.78 (dd, J^1 = 28.2, J^2=11.3 Hz, F *ortho*), -136.53 (dd, J^1 = 28.2, J^2=11.3 Hz, F *meta*) ppm.

HRMS-ESI: m/z calcd to $C_{68}H_{34}F_{16}N_8S_4$ $[M+H]^+$; 1395.1612 found: 1395.1611

3. Compounds Description: NMR

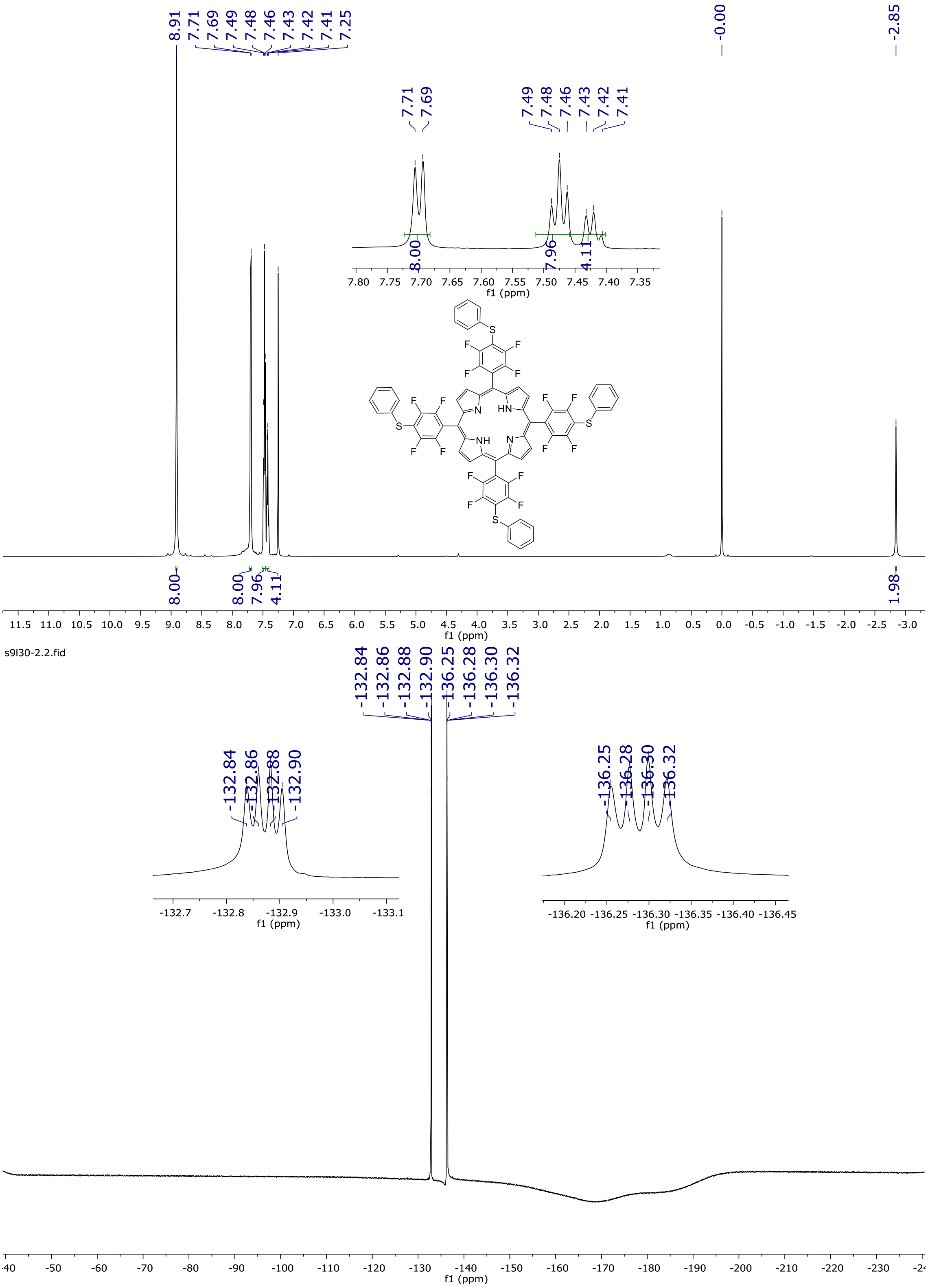

Figure S1. ^{1}H and ^{19}F NMR of the compound **3a** in $CDCl_3$ at 600 MHz and 565 MHz respectively.

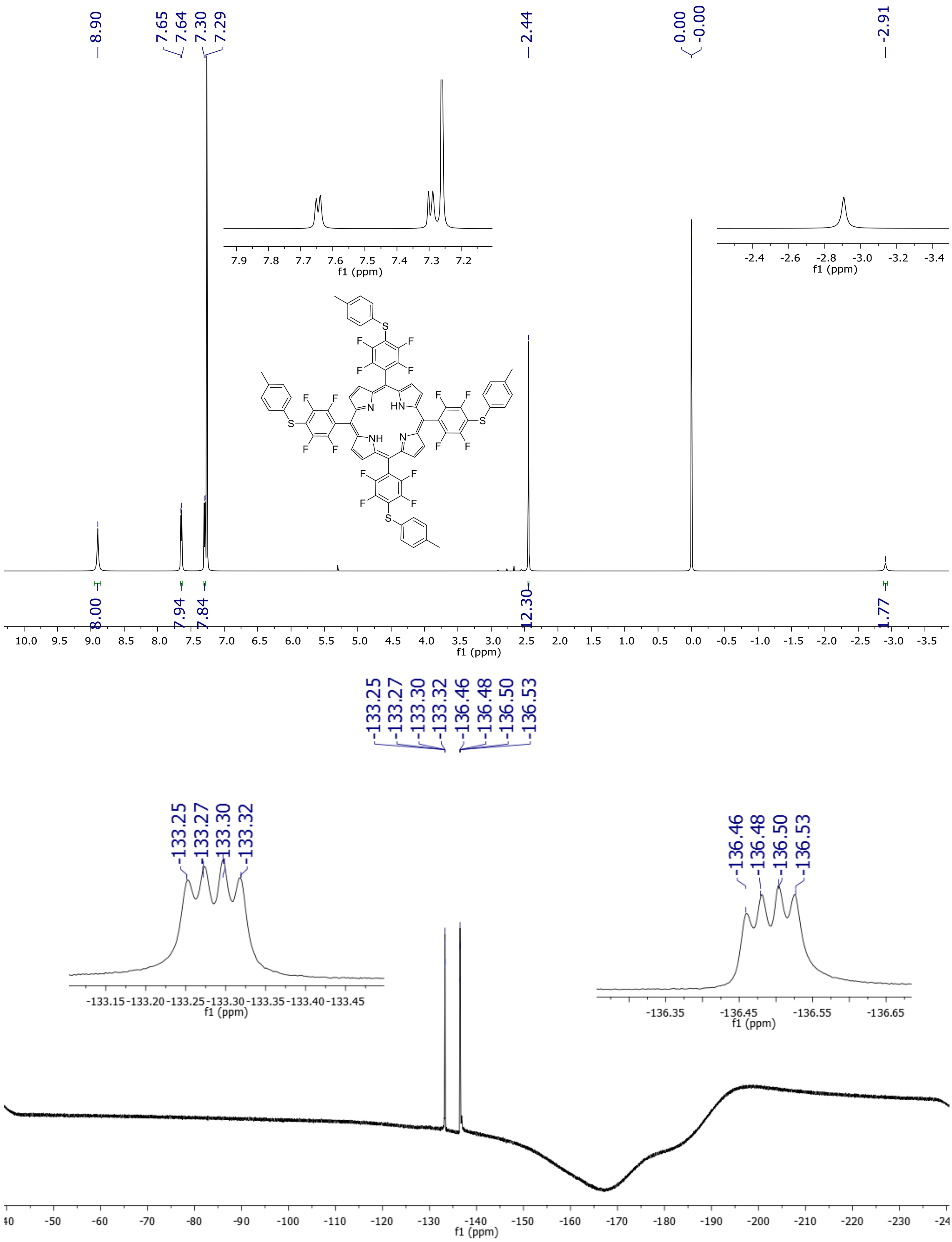

Figure S2.^{1}H, ^{19}F NMR of the compound **3b** in $CDCl_3$ at 600 MHz and 565 MHz

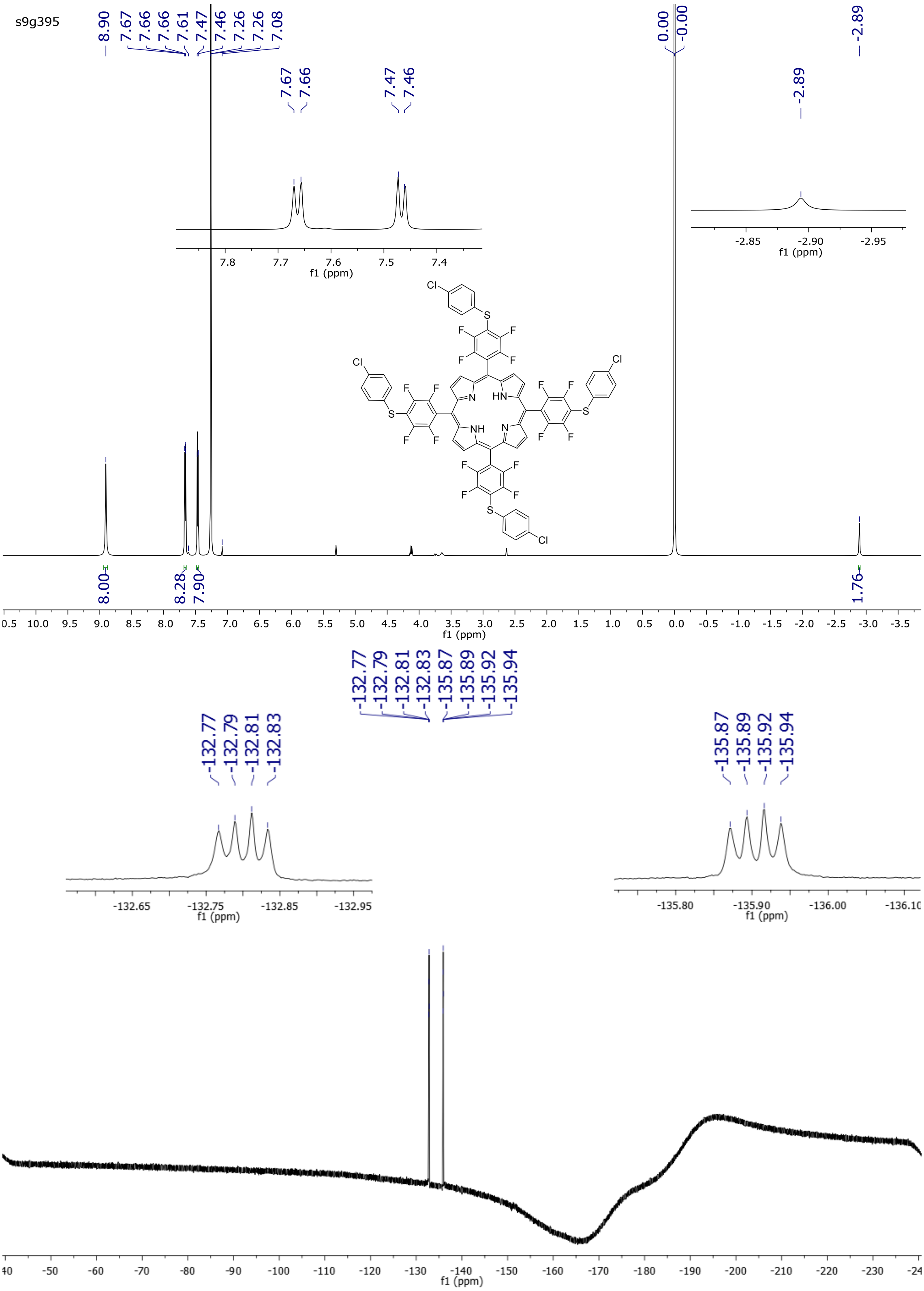

Figure S3. ^{1}H, ^{19}F NMR of the compound **3c** in $CDCl_3$ at 600 MHz and 565 MHz

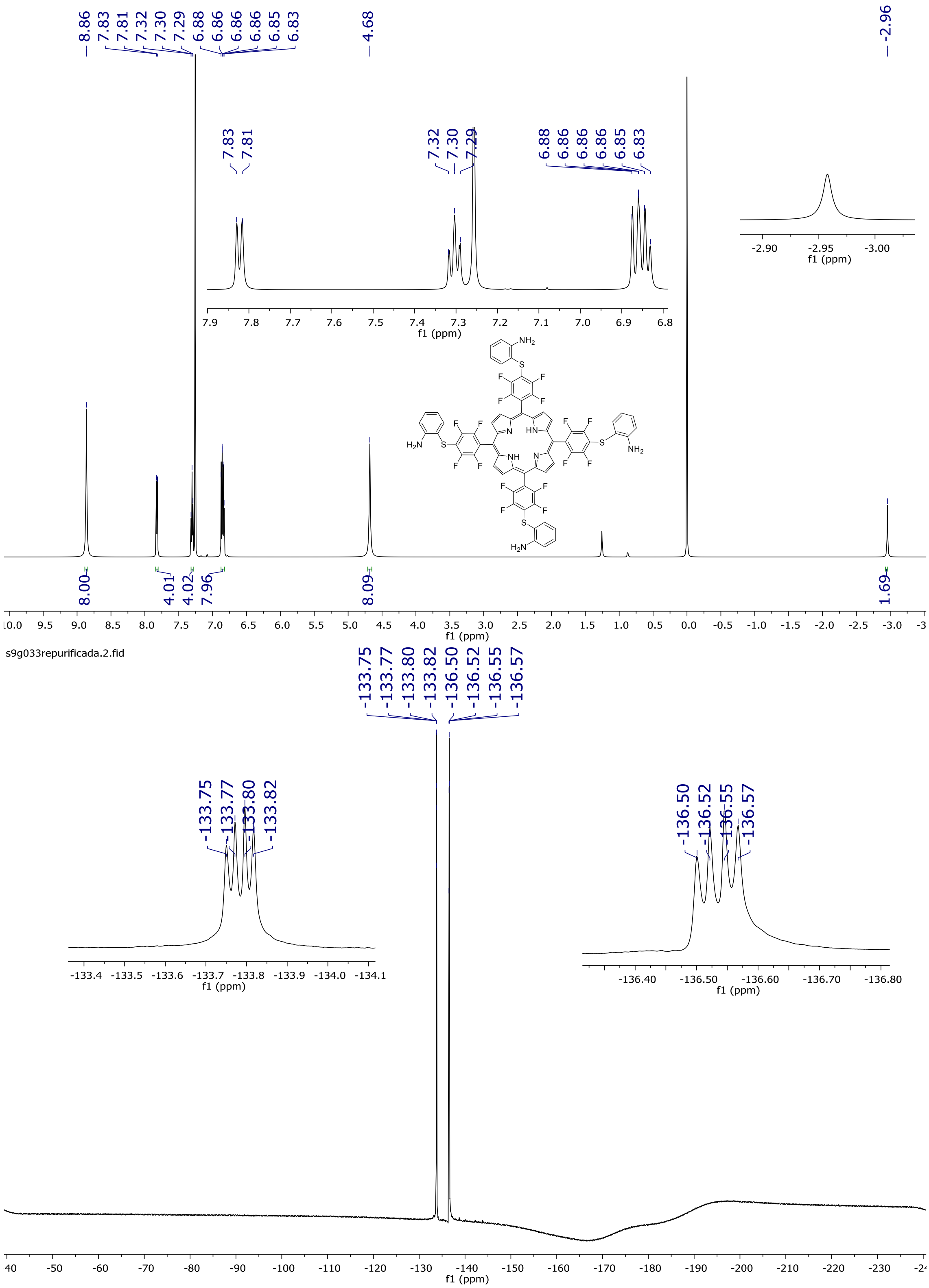

Figure S4. ^{1}H, ^{19}F NMR of the compound **3d** in $CDCl_3$ at 600 MHz and 565 MHz

4. High resolution mass spectra

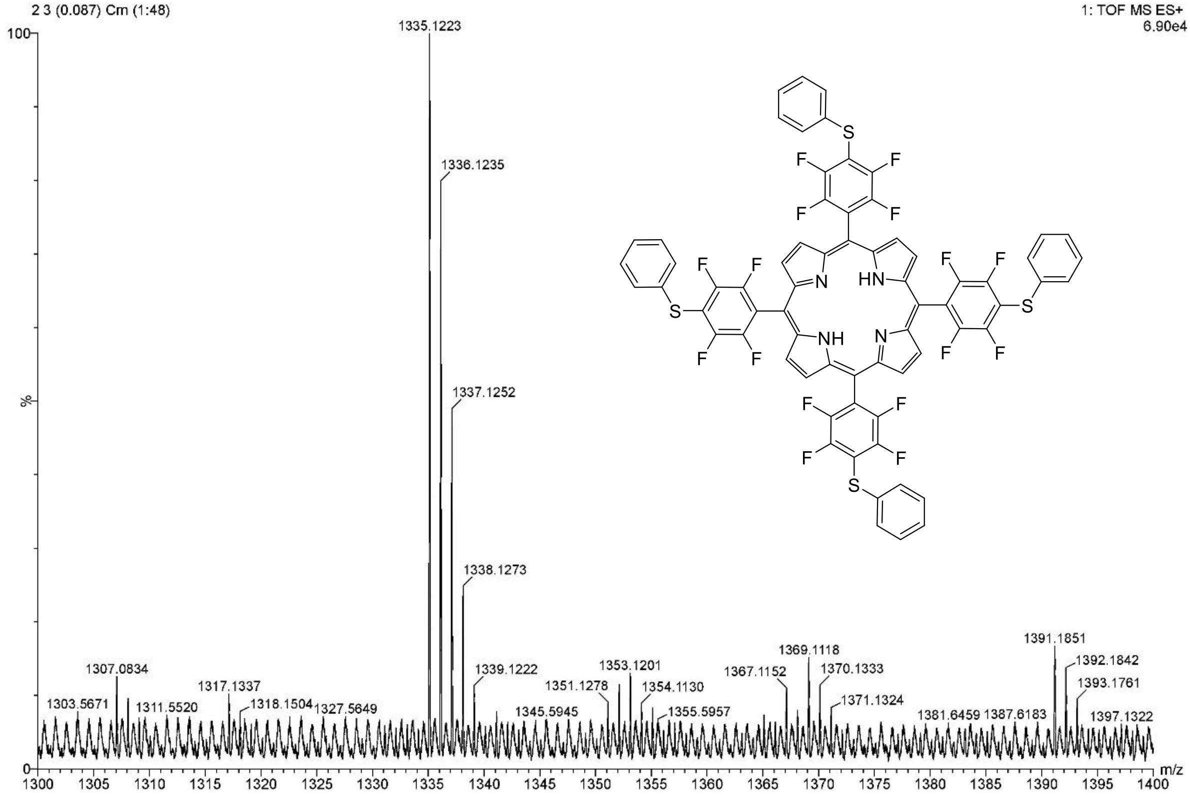

Figure S5. HRMS of the compound **3a**

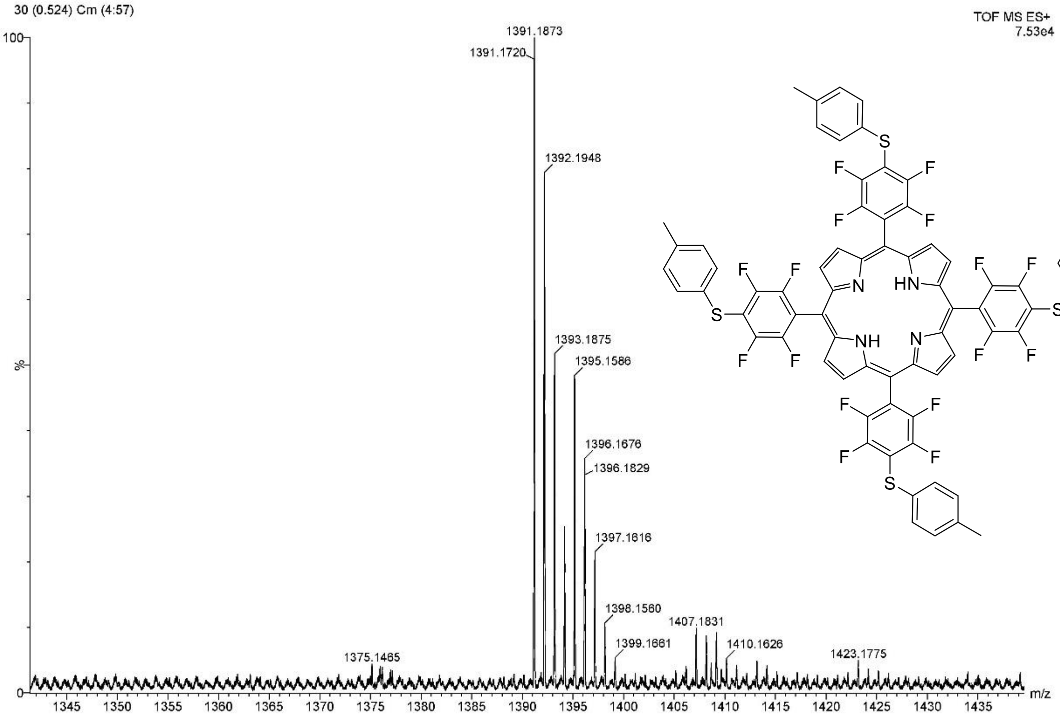

Figure S6. HRMS of the compound **3b**

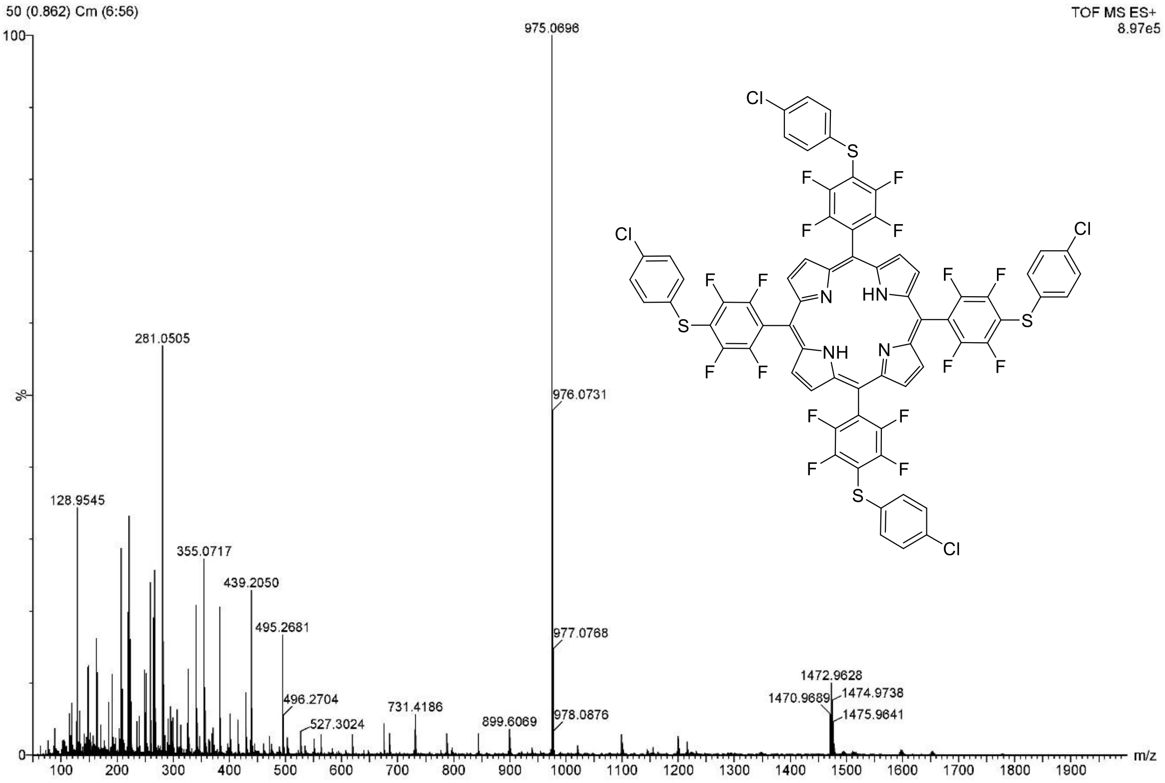

Figure S7. HRMS of the compound **3c**

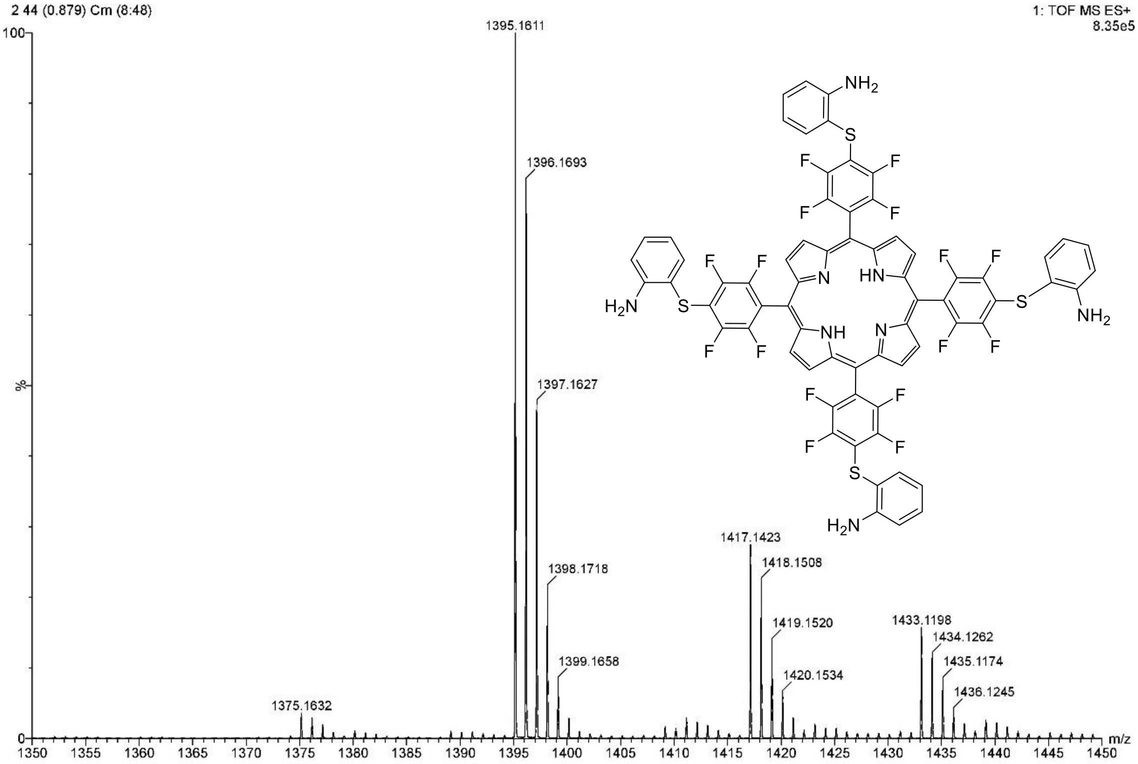

Figure S8. HRMS of the compound **3d**

5. **DNA UV-vis absorption spectra**

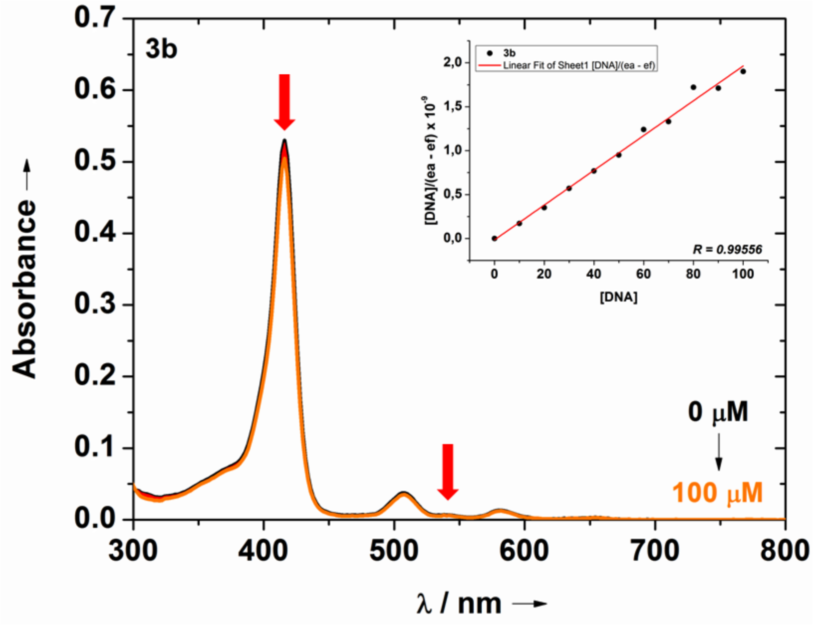

Figure S9. UV–vis titration absorption spectra of porphyrin **3b**, in a DMSO/Tris-HCl buffer (pH 7.4) mixture. The concentration of ct-DNA ranged from 0 to 100 μM. Insert graph shows the plot of [DNA]/($\varepsilon_a - \varepsilon_f$) *versus* [DNA].

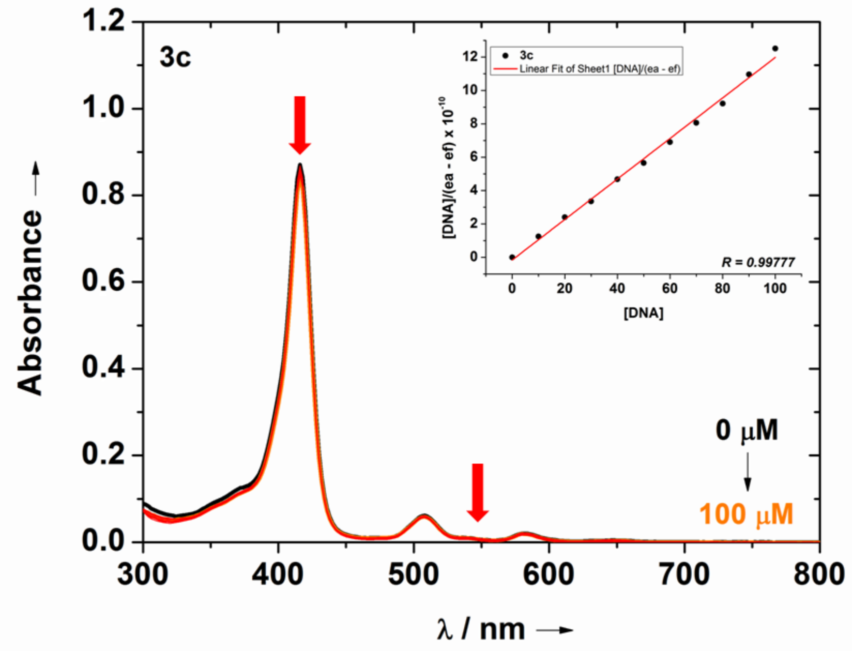

Figure S10. UV–vis titration absorption spectra of porphyrin **3c**, in a DMSO/Tris-HCl buffer (pH 7.4) mixture. The concentration of ct-DNA ranged from 0 to 100 μM. Insert graph shows the plot of [DNA]/($\varepsilon_a - \varepsilon_f$) *versus* [DNA].

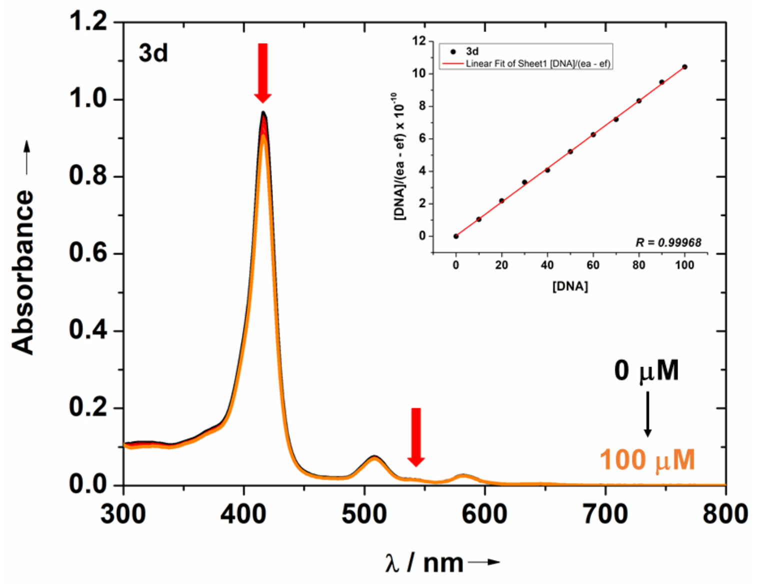

Figure S11. UV–vis titration absorption spectra of porphyrin **3d**, in a DMSO/Tris-HCl buffer (pH 7.4) mixture. The concentration of ct-DNA ranged from 0 to 100 µM. Insert graph shows the plot of [DNA]/($\varepsilon_a - \varepsilon_f$) *versus* [DNA].

6. EB-DNA emission fluorescence spectra

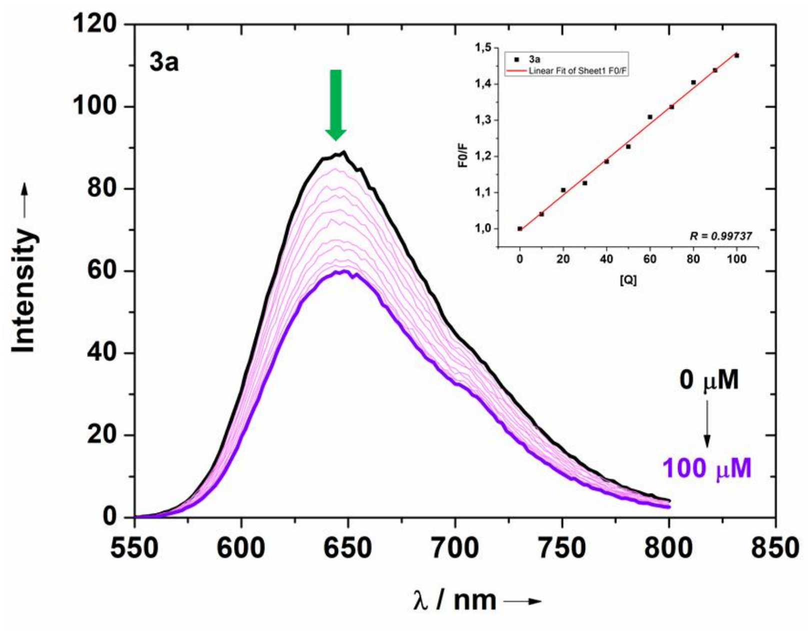

Figure S12. Emission fluorescence spectra of EB bound to ct-DNA in the presence of porphyrin **3a** in a DMSO/Tris-HCl buffer mixture at λ_{exc} = 510 nm. The arrow indicates the changes in fluorescence intensities at increasing concentrations of samples. Insert graph shows the plot of F_0/F *versus* [ct-DNA].

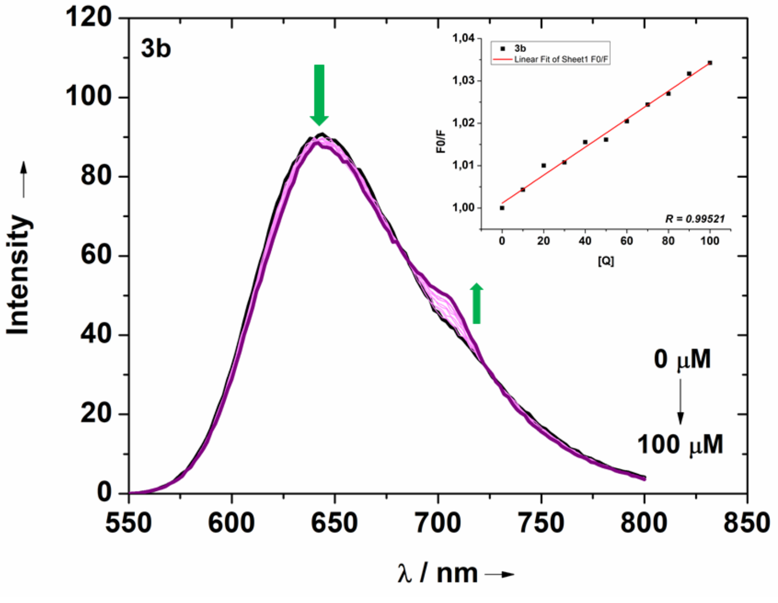

Figure S13. Emission fluorescence spectra of EB bound to ct-DNA in the presence of porphyrin **3b** in a DMSO/Tris-HCl buffer mixture at λ_{exc} = 510 nm. The arrow indicates the changes in fluorescence intensities at increasing concentrations of samples. Insert graph shows the plot of F_0/F *versus* [ct-DNA].

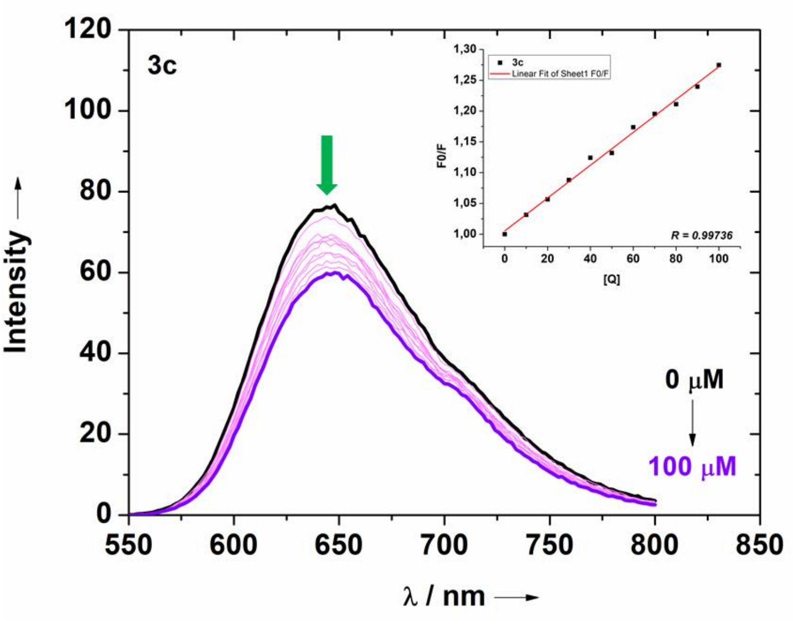

Figure S14. Emission fluorescence spectra of EB bound to ct-DNA in the presence of porphyrin **3c** in a DMSO/Tris-HCl buffer mixture at λ_{exc} = 510 nm. The arrow indicates the changes in fluorescence intensities at increasing concentrations of samples. Insert graph shows the plot of F_0/F *versus* [ct-DNA].

7. Porphyrin docking with DNA

The 3D structures of DNA were obtained from the Protein Data Bank (http://www.rcsb.org/pdb/) with the code: 423D [5]. The Chimera 1.8 software[6] was used to remove waters, ions, and other molecules, and add hydrogens to the DNA structure. The compounds **3a-d** were built in the software Avogadro 1.1.1 [7] , followed by semi-empirical PM6[8] (geometry optimization, using the program MOPAC2012 [9]. The compounds and the DNA in the *pdbqt* format were generated by AutoDockTools, where the ligands were considered flexible (with PM6 charges), and the DNA rigid (with Gasteiger charges)[10] . AutoDock Vina 1.1.1 program was used for the docking simulations[11], using a grid box of 60^3 Å (coordinates: x = -7.914, y = 52.208, z = -0.176) and an exhaustiveness of 50 and 1.0 Å of grid spacing. As a model of interactions, it was selected the conformer of lowest binding free energy. The results from the docking simulations were analyzed using the Accelrys Discovery Studio 3.5 software [12].

References

1. Iglesias, B. A., Hörner, M., Toma, H. E., Araki, K., 5-(1-(4-phenyl)-3-(4-nitrophenyl)triazene)-10,15,20- triphenylporphyrin: a new triazene-porphyrin dye and its spectroelectrochemical properties. *J. Porphyrins Phthalocyanines,* **2012,** 16, 200–209, DOI: 10.1142/S1088424612004501

2. Hadjur, C., Lange, N., Rebstein, J., Monnier, P., Van den Bergh H, Wagnières G., Spectroscopic studies of photobleaching and photoproduct formation of *meta*(tetrahydroxyphenyl)chlorin (*m*-THPC) used in photodynamic therapy. The production of singlet oxygen by *m*-THPC. *J. Photochem. Photobiol. B,* **1998**, *45,* 170–178, DOI: https://doi.org/10.1016/S1011-1344(98)00177-8

3. Auras, B. L., Meller, S. L., da Silva, M. P., Neves, A., Cocca, L. H. Z., De Boni, L., da Silveira, C. H., Iglesias, B. A., Synthesis, spectroscopic/electrochemical characterization and DNA interaction study of novel ferrocenyl-substituted porphyrins. *Appl Organometal Chem.* **2018**; *32: e4318,* 1-12, DOI: 10.1002/aoc.4318
4. Pineiro, M., Carvalho, A. L., Pereira, M. M., Gonsalves, A. M. R., Arnaut, L. G., Formosinho, S. J., Photoacoustic Measurements of Porphyrin Triplet-State Quantum Yields and Singlet-Oxygen Efficiencies. *Chem. Eur. J.* **1998**, *4*, 2299-2307, DOI: https://doi.org/10.1002/(SICI)1521-3765(19981102)4:11%3C2299::AID-CHEM2299%3E3.0.CO;2-H
5. Rozenberg, H., Rabinovich, D., Frolow, F., Hegde, R.S., Shakked, Z. Structural code for DNA recognition revealed in crystal structures of papillomavirus E2-DNA targets. *Proc. Natl. Acad. Sci. USA*, **1998**, *95*, 15194–15199, DOI: https://doi.org/10.1073/pnas.95.26.15194
6. Pettersen, E.F., Goddard, T.D., Huang, C.C., Couch, G.S., Greenblatt, D.M., Meng, E.C., Ferrin T.E., UCSF Chimera—A Visualization System for Exploratory Research and Analysis. *J. Comput. Chem*. **2004**, *25*, 1605–1162, DOI: 10.1002/jcc.20084

7. Hanwell, M.D., Curtis, D.E., Lonie, D.C., Vandermeersch, T., Zurek, E., Hutchison, G. R., Avogadro: an advanced semantic chemical editor, visualization, and analysis platform *J. Cheminform*. **2012**, *4*, 1-17, DOI: https://doi.org/10.1186/1758-2946-4-17

8. Stewart, J.J.P., Optimization of parameters for semiempirical methods V: Modification of NDDO approximations and application to 70 elements. *J. Mol. Mod*. **2007**, *13*, 1173-1213, DOI: 10.1007/s00894-007-0233-4

9. Stewart J.J.P., MOPAC2012, **2012**. Stewart Computational Chemistry. Colorado Springs, CO, USA. http://OpenMOPAC.net
10. Morris, G.M., Huey, R., Lindstrom, W., Sanner, M. F., Belew, R.K., Goodsell, D.S., Olson, A.J., Software News and Updates AutoDock4 and AutoDockTools4: Automated Docking with Selective Receptor Flexibility *J. Comput. Chem*. **2009**, *30*, 2785–2791, DOI: 10.1002/jcc.21256
11. Trott O. and Olson A.J., Software News and Update AutoDock Vina: Improving the Speed and Accuracy of Docking with a New Scoring Function, Efficient Optimization, and Multithreading. *J. Comput. Chem*. **2010**, *31*, 455-461, DOI: 10.1002/jcc.21334
12. Dassault Systèmes BIOVIA, Discovery Studio Modeling Environment, Release **2017**, San Diego: Dassault Systèmes, **2016**
